# Translational Applications of Retinal Biomarkers in Chronic Cardiovascular, Neurological, Metabolic, and Renal Diseases

**DOI:** 10.3390/vision10040078

**Published:** 2026-10-09

**Authors:** Matteo Capobianco, Marco Zeppieri, Simonetta Gaia Nicolosi, Niccolò Castellino, Francesco Cappellani, Irene Gattazzo, Caterina Gagliano

**Affiliations:** 1 Eye Clinic, University of Catania, Policlinico G. Rodolico, 95121 Catania, Italy; capobiancoteo@gmail.com; 2 Faculty of Medicine, University of Catania, 95123 Catania, Italy; 3 Department of Ophthalmology, University Hospital of Udine, 33100 Udine, Italy; 4 Department of Medicine, Surgery and Health Sciences, University of Trieste, 34129 Trieste, Italy; 5 Department of Ophthalmology, University of Catania, 95123 Catania, Italy; 6 Department of Medicine and Surgery, University of Enna “Kore”, Piazza dell’Università, 94100 Enna, Italy; 7 Mediterranean Foundation “G.B. Morgagni”, 95125 Catania, Italy; 8 Department of Ophthalmologythis, Ospedale Sant’Antonio, Azienda Ospedaliera, 35127 Padova, Italy

**Keywords:** artificial intelligence, cardiovascular diseases, diabetes mellitus, retina, tomography, optical coherence

## Abstract

Imaging the retina is a powerful non-invasive probe of systemic vascular, neurological, metabolic, and renal disease, making it a cornerstone of the new field of systemic oculomics. Advanced retinal imaging techniques like optical coherence tomography (OCT) and optical coherence tomography angiography (OCTA) provide detailed imaging of structural and microvascular changes potentially associated with systemic disease. Derived from fundus photography, structural imaging, vascular imaging, and artificial intelligence-based image analysis, retinal biomarkers have been associated with cardiovascular and cerebrovascular disease, neurodegenerative disorders, diabetes, chronic kidney disease, and related clinical outcomes. However, the translational maturity of these biomarkers is variable depending on imaging modality, target disease, intended clinical use, and level of validation. Longitudinally, retinal vascular characteristics have been associated with cardiovascular as well as cerebrovascular outcomes. In addition, optical coherence tomography has provided reproducible measures of neuroaxonal injury in selected neurological conditions. This is especially true for multiple sclerosis. The majority of retinal biomarkers in Alzheimer disease, Parkinson disease, chronic kidney disease, and systemic risk prediction remain experimental because of heterogeneous methodologies, insufficient external validation, and insufficient evidence for incremental clinical utility. Diabetes is the most developed translational model with established retinal screening pathways and validated ocular applications of imaging and artificial intelligence, although these cannot be directly extrapolated to systemic diagnosis or treatment guidance. This narrative review assesses retinal biomarkers for cardiovascular, neurological, metabolic, and renal disease with a focus on analytical validity, clinical validity, incremental value, clinical utility, and implementation. The retina is increasingly accepted as a site for discovery of systemic biomarkers and adjunctive risk assessment, although most applications to systemic disease remain to be standardized, validated independently, and studied prospectively before being suitable for routine clinical use.

## 1. Introduction

Diabetes provides a clinically informative model of the convergence of metabolic, vascular, and neurological injury. A pooled analysis of 1108 population-representative studies comprising 141 million adults aged ≥18 years estimated that 828 million people (95% credible interval [CrI], 757–908 million) were living with diabetes in 2022, representing an increase of 630 million (95% CrI, 554–713 million) since 1990. Among adults aged ≥30 years with diabetes, 445 million (95% CrI, 401–496 million), corresponding to 59% of this population, were not receiving glucose-lowering medication. Both the increase in prevalence and the treatment deficit were concentrated predominantly in low- and middle-income countries [1]. The ocular burden is similarly substantial. A meta-analysis of 59 population-based studies estimated that, among individuals with diabetes, the global prevalence of diabetic retinopathy was 22.27% (95% confidence interval [CI], 19.73–25.03%), whereas vision-threatening diabetic retinopathy and clinically significant macular edema affected 6.17% (95% CI, 5.43–6.98%) and 4.07% (95% CI, 3.42–4.82%), respectively. The corresponding numbers of affected adults were estimated at 103.12, 28.54, and 18.83 million in 2020 and are projected to reach 160.50, 44.82, and 28.61 million by 2045. This burden is geographically heterogeneous, with diabetic retinopathy prevalence ranging from 13.37% in South and Central America to 35.90% in Africa [2]. Retinal disease is therefore not a marginal complication of diabetes but a frequent and unevenly distributed manifestation occurring within the broader systemic burden of vascular and metabolic injury.

The translational value of the retina arises from the combination of its biological relevance and its accessibility to repeated, non-invasive imaging. This premise underlies oculomics, defined as the application of ophthalmic biomarkers to investigate systemic disease mechanisms and support disease detection and prediction. Color fundus photography captures both qualitative abnormalities, including retinopathy signs, and quantitative vascular characteristics, such as vessel caliber and tortuosity. Optical coherence tomography provides depth-resolved measurement of retinal architecture and individual retinal-layer thicknesses, whereas optical coherence tomography angiography (OCTA) enables layer-specific assessment of perfused vascular networks and capillary density. These directly interpretable measurements differ conceptually from image-derived signatures learned by artificial intelligence (AI) models, which may incorporate patterns that are not predefined or readily perceptible to human observers [3,4,5]. The biological basis for their systemic relevance is particularly strong for neurological disease: the retina originates from the diencephalon and constitutes an extension of the central nervous system, sharing neurons, axons, glial components, specialized immune responses, and a blood–retinal barrier that closely resembles the blood–brain barrier [6]. Nevertheless, an association or accurate prediction derived from a retinal image does not, by itself, demonstrate a causal mechanism, validate a surrogate endpoint, or establish clinical benefit. Current AI evidence remains constrained by limited prospective and longitudinal data, under-representation of rare or complex diseases, heterogeneous image acquisition, dependence on predominantly single-modality datasets, and risks of dataset bias and limited interpretability. Retinal features and AI-derived signatures should therefore be regarded as candidate systemic biomarkers requiring standardized acquisition, independent validation in diverse populations, and prospective evaluation against established clinical measures and patient-relevant outcomes before routine implementation [3,7].

The evidence base has not matured uniformly. Color fundus photography is supported by longitudinal cohorts linking vessel caliber and retinopathy signs to incident hypertension, stroke, coronary events, and mortality; however, the reported coronary associations vary according to age and sex. In 16-year follow-up data from the Atherosclerosis Risk in Communities study, narrower retinal arterioles and wider venules were associated with stroke and mortality, while adding retinal vessel diameters to conventional assessment reclassified 21% of women initially considered to have a <5% 10-year cardiovascular risk into an intermediate-risk category [3]. OCT is established in ophthalmic care and enables quantitative neuro-ophthalmic assessment, whereas its application as a systemic prognostic biomarker still requires stronger longitudinal confirmation [3,6]. OCTA provides depth-resolved, dye-free visualization of perfused capillary plexuses but cannot assess vascular leakage or very slow blood flow, and interoperability, acquisition protocols, segmentation, and image-quality requirements remain insufficiently standardized across devices and studies [3,4]. Regulatory status is jurisdiction-specific and should be distinguished from evidence of clinical utility. In the United States, IDx-DR received FDA De Novo authorization for diabetic retinopathy detection in 2018, and EyeArt subsequently received FDA 510(k) clearance for the same general clinical application. SELENA+ received regulatory approval from the Singapore Health Sciences Authority in 2019 for AI-assisted detection of diabetic retinopathy and other ocular conditions. Among systemic oculomics applications, Reti-CVD, currently marketed as Dr.Noon CVD, has received regulatory authorization in specific jurisdictions for cardiovascular risk assessment. However, such regulatory authorization establishes permission for the specified intended use within the relevant jurisdiction and should not be interpreted as evidence that retinal AI-guided cardiovascular assessment improves clinical decision-making or patient outcomes.

**Figure 1:**
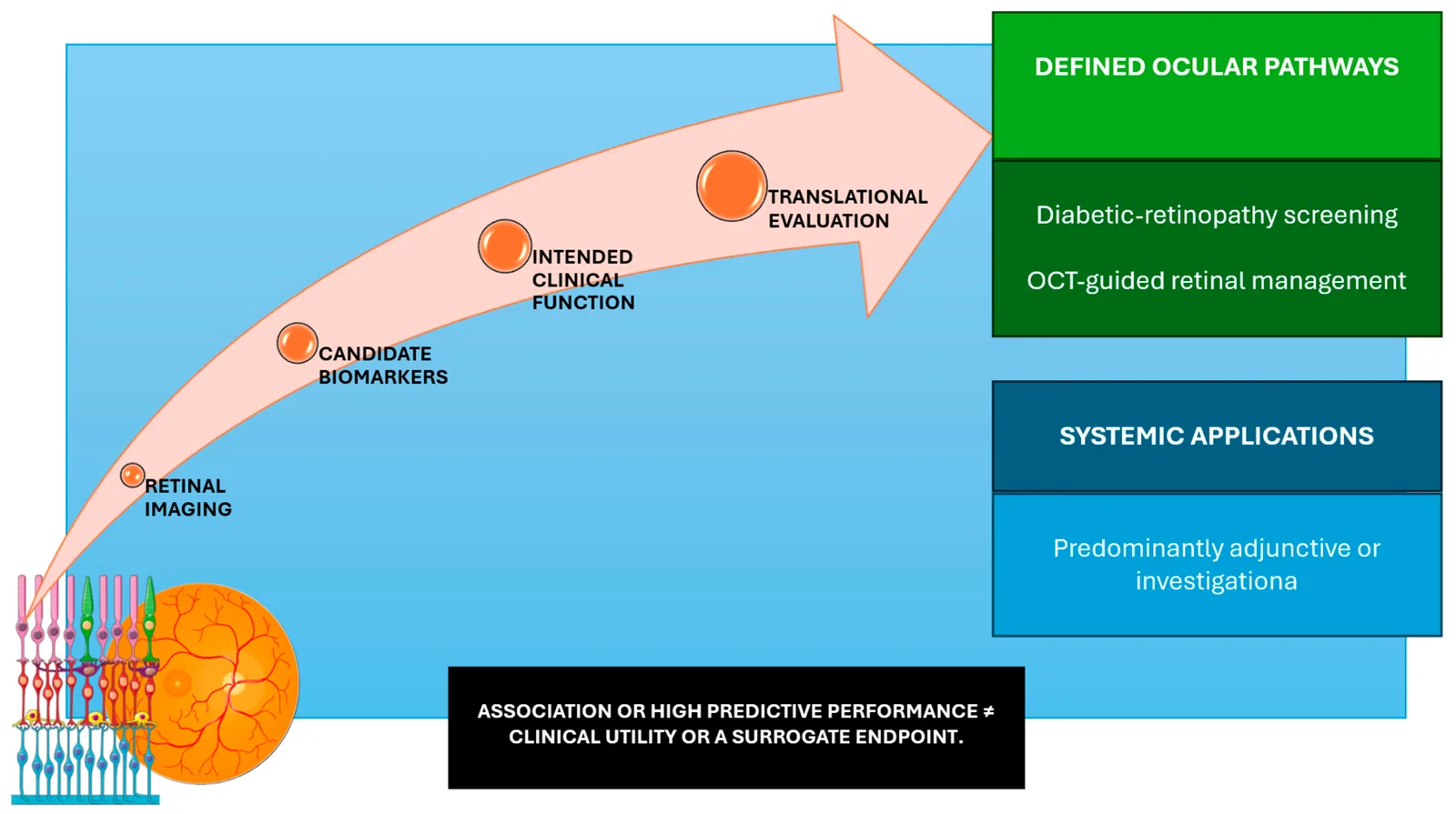
Use-case-specific translational pathway for retinal biomarkers. Retinal measurements and AI-derived signatures must be evaluated according to their intended clinical function and independently satisfy requirements for analytical validity, clinical validity, incremental value, clinical utility, and implementation. Defined clinical pathways currently remain predominantly ocular, whereas most cardiovascular, neurological, metabolic, and renal applications are adjunctive or investigational. AI, artificial intelligence; OCT, optical coherence tomography; OCTA, optical coherence tomography angiography. Image provided by Servier Medical Art (https://smart.servier.com), licensed under CC BY 4.0 (accessed on 31 August 2026, https://creativecommons.org/licenses/by/4.0/).

Against this background, this narrative review critically appraises retinal biomarkers across chronic cardiovascular and cerebrovascular, neurological and neurodegenerative, metabolic, and renal diseases. Evidence is organized according to the intended biomarker function—detection, diagnosis, prognosis, or monitoring of disease and treatment response—and evaluated in terms of biological plausibility, analytical performance, validation against appropriate reference standards, incremental value over established clinical pathways, and feasibility of implementation. Throughout this review, ‘biomarker’ is used as an overarching term, whereas specific functional terminology is used when supported by the evidence: prognostic biomarkers indicate the likelihood of future clinical outcomes or disease progression, predictive biomarkers identify differential effects of an intervention, pharmacodynamic biomarkers indicate a biological response following an intervention, and surrogate endpoints are biomarkers used as substitutes for direct measures of clinical benefit. Diabetes serves as the index condition because it combines a common retinal complication with established screening programs and regulated AI-assisted diagnostic pathways. Comparisons with other chronic disorders are used to distinguish retinal findings that may reflect shared systemic processes from disease-specific manifestations and from associations that remain insufficiently validated or clinically non-actionable [2,4]. The use-case-specific pathway from retinal image acquisition to clinical implementation is summarized in Figure 1.

## 2. Materials and Methods

This study was designed as a critical narrative review. The preparation of this review was guided by the six domains of the Scale for the Assessment of Narrative Review Articles (SANRA): justification of the review’s importance, explicit statement of its aims, description of the literature search, appropriate referencing, scientific reasoning, and presentation of relevant endpoint data. SANRA was used to support the quality and transparency of manuscript preparation. It was not used as a reporting guideline or as an instrument for assessing risk of bias in the included studies [8].

We conducted a literature search in PubMed from database inception through September 2026. Search terms covered retinal and ophthalmic imaging, systemic disease domains, and clinically relevant outcomes or analytical approaches. A representative PubMed search string was as follows: (“retina”[Title/Abstract] OR “retinal imaging”[Title/Abstract] OR “fundus photography”[Title/Abstract] OR “retinal vessel caliber”[Title/Abstract] OR “retinal vessel calibre”[Title/Abstract] OR “retinopathy”[Title/Abstract] OR “optical coherence tomography”[Title/Abstract] OR “OCT”[Title/Abstract] OR “optical coherence tomography angiography”[Title/Abstract] OR “OCTA”[Title/Abstract] OR “oculomics”[Title/Abstract] OR “retinal biomarker”[Title/Abstract] OR “retinal biomarkers”[Title/Abstract]) AND (“cardiovascular disease”[Title/Abstract] OR “hypertension”[Title/Abstract] OR “coronary disease”[Title/Abstract] OR “stroke”[Title/Abstract] OR “dementia”[Title/Abstract] OR “Alzheimer disease”[Title/Abstract] OR “Parkinson disease”[Title/Abstract] OR “multiple sclerosis”[Title/Abstract] OR “diabetes”[Title/Abstract] OR “chronic kidney disease”[Title/Abstract] OR “mortality”[Title/Abstract] OR “artificial intelligence”[Title/Abstract] OR “deep learning”[Title/Abstract]). Because this was a narrative review, the search syntax was iteratively adapted to individual disease domains, retinal biomarker modalities, and specific clinical outcomes to identify additional relevant literature, rather than being applied as a single fixed systematic-review query. No additional PubMed search filters were applied; study eligibility was determined according to the criteria described below. Given the narrative rather than systematic design of the review, PubMed was selected as the primary bibliographic database because of its broad coverage of biomedical and clinical literature across the disciplines considered.

Eligible studies were English-language, peer-reviewed human studies evaluating a retinal structural, vascular, functional, or computationally derived feature in relation to a chronic systemic disease, a clinically relevant systemic outcome, or its use as part of a retinal screening or prediction pathway. Interpretive priority was given to systematic reviews, meta-analyses, consensus or expert-roadmap documents, large longitudinal cohorts, externally validated prediction models, and prospective implementation studies, particularly those published from 2019 onward. Earlier studies remained included whenever they established a landmark association, offered a measurement framework, or provided longitudinal evidence that remained essential for interpreting later work. Excluded studies comprised case reports, non-peer-reviewed preprints, animal-only investigations, and studies restricted to local ocular disease without a systemic or translational endpoint. Purely technical proof-of-concept studies were not prioritized unless they incorporated external validation or addressed a clinically defined use case. Reference lists of major reviews and consensus documents were also examined to identify additional key studies, and bibliographic information, PubMed indexing, and DOI identifiers were confirmed for all references.

For evidence synthesis, studies were grouped according to imaging modality, biomarker domain, systemic disease, study design, clinical endpoint, setting of validation, and translational maturity. Whenever available, quantitative data—including sample size, follow-up duration, effect estimates with uncertainty intervals, and diagnostic or predictive performance measures—were retained rather than reduced to qualitative descriptions. Due to substantial differences in their population characteristics, imaging devices, acquisition protocols, segmentation algorithms, definitions of retinal features, reference standards, and clinical outcomes, a new meta-analysis was not undertaken. The narrative synthesis gives more interpretive weight to consistency across independent studies, temporal evidence from longitudinal cohorts, external and prospective validation, comparison with established clinical predictors, and demonstrated relevance to patient care. As this was a narrative rather than a systematic review, exhaustive retrieval of all eligible studies was not intended, and no formal study-level risk-of-bias assessment was performed.

## 3. Results

### 3.1. Retinal Biomarker Domains and Measurement Architecture

Within this measurement architecture, diabetic retinopathy provides the reference model because diabetes affects the retinal neurovascular unit rather than an isolated vascular compartment. Hyperglycemia-associated metabolic and oxidative stress disrupts signaling among neurons, glial cells, endothelial cells, and pericytes. Reactive gliosis and neuronal apoptosis, impaired neurovascular coupling, endothelial tight-junction failure, pericyte loss, inflammatory activation, and VEGF-mediated permeability therefore interact and may precede conventionally visible retinopathy. The resulting biomarkers represent different levels of the same pathological system: color fundus photography records visible lesions—including microaneurysms, hemorrhages, venous beading, intraretinal microvascular abnormalities, and neovascularization—as well as vascular geometry; OCT quantifies retinal and choroidal anatomy, layer-specific neuroretinal changes, and edema; and OCTA characterizes detected perfusion within anatomically separated capillary plexuses [9,10,11]. These domains are biologically connected but are neither technically nor clinically interchangeable.

This distinction is particularly important for OCTA. An OCTA image is a motion-contrast map constructed from changes in the OCT signal produced by moving blood cells. Vessel or perfusion density represents the area occupied by detected perfused vasculature within a defined region, rather than a direct measurement of volumetric blood flow. OCTA also does not demonstrate vascular leakage, while flow below the detection threshold may appear as non-perfusion. Even apparently intuitive measures require caution: FAZ area varies by approximately threefold between individuals, with reported values in healthy eyes ranging from 0.110 to 0.861 mm^2^, limiting the validity of a single cross-sectional diagnostic threshold [12]. Methodological heterogeneity compounds this biological variability. A systematic review of 191 studies identified seven OCTA devices, six macular volume dimensions, four macular subregions, nine perfusion-analysis approaches, and five definitions of vascular layers, producing 197 methods actually used to assess macular perfusion and more than 7000 theoretically possible combinations. Absolute results were therefore not directly comparable across many platforms and analytical pipelines [13]. For structural OCT, APOSTEL 2.0 used a modified Delphi process involving 116 participating authors: 12 of 15 proposed revisions were incorporated into an updated nine-item checklist, with agreement exceeding 90%. The recommendations require reporting of acquisition protocols, measurement areas, device-specific features, segmentation procedures, and potential bias from manual correction. Importantly, OCTA was deliberately excluded because standardized quantitative metrics and quality-control criteria were considered insufficiently established [14].

Evidence for abnormalities before clinically visible diabetic retinopathy is consequently persuasive at the group level but insufficient for individual classification. A meta-analysis of 36 studies and one additional cohort, comprising 6220 participants, found modest thinning in eyes with diabetes without retinopathy compared with controls for macular ganglion cell–inner plexiform layer thickness (standardized mean difference [SMD], −0.26; 95% CI, −0.42 to −0.11; I^2^ = 62%), macular retinal nerve fiber layer (RNFL) thickness (SMD, −0.26; 95% CI, −0.51 to −0.01; I^2^ = 52%), and macular ganglion cell complex thickness (SMD, −0.28; 95% CI, −0.48 to −0.09; I^2^ = 46%). Conversely, mean peripapillary RNFL thickness did not differ significantly from controls, and only one eligible longitudinal study was identified [15]. Similarly, an OCTA meta-analysis of 44 cross-sectional studies involving 2221 diabetic and 1838 healthy eyes found larger FAZ measurements and lower perfusion density across several macular and peripapillary regions before visible retinopathy. However, most differences became non-significant in type 1 diabetes subgroup analyses, whereas they generally persisted in type 2 diabetes, indicating dependence on population and disease characteristics [16]. These findings establish detectable structural and perfusion signals but not a universal preclinical threshold. Multimodal interpretation is therefore more defensible than classification based on an isolated metric, while AI-derived representations should be regarded as an analytical layer built upon these inputs rather than as independent biological measurements capable of overcoming image-quality or measurement limitations [17].

### 3.2. Heart and Brain Diseases

As seen through retinal photography, a systemic vascular signal can be identified by longitudinal evidence. However, its magnitude and clinical significance will vary with respect to the retinal phenotype and cardiovascular outcome being examined. Over a mean follow-up of 3.5 years in the ARIC cohort, 110 incident strokes occurred among 10,358 participants. After adjustment for conventional risk factors, any retinopathy was associated with stroke (RR, 2.58; 95% CI, 1.59–4.20), with similar associations reported for microaneurysms, soft exudates, and blot hemorrhages [18]. The coronary association was more sex-specific. In 9648 ARIC participants without a history of coronary heart disease (CHD), a one-standard-deviation decrease in the arteriole-to-venule ratio was associated with incident CHD in women (RR, 1.37; 95% CI, 1.08–1.72) but not in men (RR, 1.00; 95% CI, 0.84–1.18) after multivariable adjustment. A decrease in one standard deviation in the arteriole-to-venule ratio was associated with incident CHD in women (RR, 1.37; 95% CI, 1.08–1.72) but not in men (RR, 1.00; 95% CI, 0.84–1.18) after adjusting for antecedent blood pressure, diabetes, smoking, lipid levels and other cardiovascular variables [19].

Separate analyses of arteriolar and venular caliber in subsequent analyses further sharpened the links. Over 5–14 years, there were 2219 incident CHD events in 22,159 participants across six cohorts. An increase in venular caliber by 20 µm was associated with CHD in women, with a hazard ratio of 1.16 (95% CI, 1.06–1.26). A decrease in arteriolar caliber by 20 µm was also associated with CHD in women (HR, 1.17; 95% CI, 1.07–1.28). Neither association was present in men [20]. On the other hand, there were 945 strokes during 5–12 years’ follow-up among 20,798 stroke-free participants at initial assessment. Wider venules predicted stroke (HR per 20 µm increase, 1.15; 95% CI, 1.05–1.25), whereas arteriolar caliber did not (HR per 20 µm decrease, 1.00; 95% CI, 0.92–1.08) [21].

Incorporating venular caliber reclassified 10.1% of participants at intermediate risk, mostly toward a lower-risk group, but this analysis did not assess whether reclassification affected treatment or outcomes. Similarly, temporality was also demonstrated for hypertension among 10,229 adults without hypertension, diabetes, or cardiovascular disease, 2599 of whom developed hypertension over median follow-up periods of 2.9–10 years. Every 20 µm reduction in arteriolar diameter was associated with an odds ratio (OR) of 1.29 (95% CI, 1.20–1.39), and every 20 µm increase in venular diameter with an OR of 1.14 (95% CI, 1.06–1.23); additionally, a 20 µm narrower baseline arteriolar diameter was associated with a 1.12-mmHg (95% CI, 0.25–1.99) greater increase in systolic blood pressure over five years [22].

The extended ARIC follow-up verified that associations were specific to the outcome. Of a total of 10,470 participants who were followed for a mean of 16 years, there were 1779 CHD events; 548 ischemic strokes; 1395 heart-failure events; and 2793 deaths. After making adjustments for variables in the Pooled Cohort Equations, the HRs for stroke were found to be 1.14 (95% CI, 1.03–1.26) per standard-deviation decrease in arteriolar caliber and 1.18 (95% CI, 1.07–1.31) per standard-deviation increase in venular caliber. Links to CHD were confined to women, while those with heart failure were lost after systolic blood pressure adjustment [23]. A meta-analysis involving five studies reported HRs for stroke of 1.14 (95% CI, 1.05–1.23) for narrower arterioles and 1.20 (95% CI, 1.12–1.28) for wider venules, both per standard-deviation change [24]. The earlier individual-participant analysis reports a null arteriolar estimate, but the later aggregation gives a positive estimate. This suggests the arteriolar association with stroke is less homogeneous than the venular association and remains sensitive to the included evidence and analytical framework.

The prespecified vascular measurements from the prior work are extended by deep learning to image-wide prediction, but whether it offers additional value compared to clinical models already established has yet to be evaluated. Importantly, predictive association did not necessarily translate into incremental value over established clinical models. In UK Biobank validation, a retinal deep-learning model achieved an AUC of 0.70 (95% CI, 0.65–0.74) for five-year major adverse cardiovascular events, compared with 0.72 (95% CI, 0.67–0.76) for SCORE; combining the retinal model with SCORE did not improve the AUC beyond 0.72 [25]. The system, which was designed to automate the measurement of retinal-vessel caliber, has shown high correlation with expert measurements when tested on more than 70,000 images. In the Singapore Epidemiology of Eye Diseases cohort, there were 851 cardiovascular events over a median of 4.54 years. In this cohort, narrower AI-measured arteriolar caliber remained associated with incident cardiovascular disease after multivariable adjustment (HR per standard-deviation decrease, 1.12–1.13, depending on the measurement zone) [26].

RetiCAC translated retinal photographs into an estimate of coronary artery calcium and, when added to the Pooled Cohort Equation, improved the C statistic by 0.031 for SCORE-defined cardiovascular events and by 0.014 for atherosclerotic cardiovascular events [27]. Similarly, adding Reti-CVD to QRISK3 increased the C statistic by 0.013–0.023 across the examined subgroups [28]. These findings indicate modest incremental predictive value, but the Reti-CVD thresholds and combined models were optimized or fitted within the same UK Biobank analysis. More importantly, neither analysis demonstrated that retinal-model–guided assessment improves clinical decisions or patient outcomes. Thus, incremental discrimination should not be equated with clinical utility.

### 3.3. Neurological and Neurodegenerative Disease

The neurological evidence must be interpreted according to the reference standard and intended biomarker function. In a systematic review, 26 cross-sectional studies associated retinal microvascular abnormalities with dementia (odds ratios [ORs], 1.17–5.57), modest cognitive deficits in individuals without dementia (effect sizes, −0.25 to 0.03), and cerebral atrophy or vascular lesions (ORs, 0.94–2.95). However, nine longitudinal studies found no consistent association with dementia or cognitive decline over 3–15 years (ORs or hazard ratios, 0.77–1.55), although prospective associations with brain-imaging abnormalities were reported [29]. Retinal microvascular findings may therefore inform the study of shared cerebral vascular injury, but the evidence does not support their use as independent diagnostic markers of neurodegeneration [29,30].

This distinction is particularly important in Alzheimer disease (AD). An umbrella review of 14 systematic reviews found that only four were at low risk of bias across all assessed domains. The most extensively investigated retinal parameters showed only poor-to-moderate discrimination between clinically diagnosed AD and healthy controls: peripapillary retinal nerve fiber layer (pRNFL) thickness had a derived area under the curve (AUC) of 0.70 (95% confidence interval [CI], 0.53–0.79; 38 studies, 1883 patients and 2510 controls), whereas foveal avascular zone area had an AUC of 0.73 (95% CI, 0.50–0.89; five studies, 177 patients and 371 controls). Because these AUCs were derived from standardized mean differences rather than prospectively validated diagnostic thresholds, they quantify group separation but do not establish an applicable clinical test [31]. When AD was instead defined by cerebral amyloid-β status, a review of 38 studies involving 2404 participants found only weak evidence for pRNFL thinning (11 studies, *n* = 828; *p* = 0.14) and foveal avascular zone enlargement (four studies, *n* = 207; *p* = 0.18); reduced arteriolar fractal dimension was more consistently observed but was supported by only three studies comprising 297 participants. No diagnostic-accuracy parameter for an individual retinal biomarker was reported by more than one study, preventing pooled evaluation of sensitivity or specificity [32].

OCTA studies reinforce the presence of a group-level vascular phenotype without resolving this diagnostic limitation. Compared with controls, superficial capillary plexus vessel density was lower in both AD (standardized mean difference [SMD], −0.48; 95% CI, −0.70 to −0.27) and mild cognitive impairment (MCI; SMD, −0.42; 95% CI, −0.81 to −0.03), while deep plexus density was also reduced and the foveal avascular zone was enlarged in AD. Device type and macular scan size significantly influenced these differences, indicating that the observed effect cannot be separated from acquisition methodology [33]. In cognitively healthy populations, 17 studies comprising 601 participants with positive AD-related biomarkers and 881 controls reported trends towards RNFL and ganglion-cell thinning and altered vascular density, but the pooled RNFL and ganglion cell–inner plexiform layer findings remained inconclusive. Moreover, “biomarker positivity” encompassed heterogeneous definitions, including amyloid or tau measurements and genetic risk variants [34]. Eye-AD, an OCTA-based deep-learning model, achieved external AUCs of 0.9007 for early-onset AD and 0.8037 for MCI [35]. These estimates came from selected Chinese case–control datasets; however, they used clinical rather than uniform amyloid-based disease definition. The authors also acknowledged limited ethnic diversity, relatively small samples, possible selection bias from excluding non-cooperative patients, and the need for longitudinal validation.

Structural OCT abnormalities are not confined to AD. A meta-analysis of 36 observational studies comprising 1712 patients with Parkinson disease and 1778 controls found mean pRNFL thinning of 3.51 μm (95% CI, 2.18–4.84 μm; I^2^ = 78%) and macular ganglion-cell-complex thinning of 4.18 μm (95% CI, 2.29–6.07 μm; I^2^ = 71.4%). Most studies were case–control, and insufficient true-positive, false-positive, true-negative, and false-negative data precluded assessment of individual diagnostic accuracy [36]. In multiple sclerosis, the neuroaxonal signal was larger and explicitly dependent on previous optic neuritis. Across 40 studies meeting OCT quality criteria, including 5776 eyes with multiple sclerosis and 1697 control eyes, pRNFL thinning was 20.10 μm (95% CI, 17.44–22.76 μm) in eyes with previous optic neuritis and 7.41 μm (95% CI, 5.83–8.98 μm) in eyes without optic neuritis. Corresponding ganglion cell–inner plexiform layer differences were 16.42 μm (95% CI, 13.60–19.23 μm) and 6.31 μm (95% CI, 4.87–7.75 μm), respectively [37].

More recent diagnostic recommendations have expanded the clinical role of OCT in multiple sclerosis. The 2024 revisions of the McDonald criteria recognize the optic nerve as an additional anatomical location for dissemination in space. Provided that rigorous quality control is applied and that there is no better alternative explanation for optic nerve involvement, an inter-eye difference of ≥6 μm in pRNFL thickness or ≥4 μm in macular GCIPL thickness can support evidence of optic nerve injury. OCT therefore has an adjunctive diagnostic role within an established neurological assessment, in addition to its role in monitoring neuroaxonal injury, but it should not be interpreted as a standalone screening or diagnostic test for multiple sclerosis [38].

Population-level associations remain less specific. Among 32,038 UK Biobank participants without reported diabetes, ocular disease, or neurological disease, thinner RNFL was associated with poorer baseline cognitive performance. Nevertheless, only 1251 participants underwent cognitive reassessment approximately three years later; compared with the thickest RNFL quintile, the two thinnest quintiles were associated with worsening on at least one cognitive test, with ORs of 1.92 (95% CI, 1.29–2.85) and 2.08 (95% CI, 1.40–3.08), respectively [39]. This outcome indicates risk of cognitive change but cannot identify its underlying pathology. Similarly, a meta-analysis of 13 OCTA studies pooling different neurodegenerative and neurovascular disorders found reduced superficial (SMD, −0.60; 95% CI, −0.78 to −0.43; I^2^ = 43%) and deep capillary plexus density (SMD, −0.84; 95% CI, −1.15 to −0.53; I^2^ = 78%) and an enlarged foveal avascular zone (SMD, 0.62; 95% CI, 0.29–0.94; I^2^ = 83%) [40]. The observational designs, moderate risk of bias, high heterogeneity, and pooling of biologically different disorders indicate a shared neurovascular signature rather than a disease-specific diagnostic phenotype. Accordingly, retinal imaging currently has its strongest neurological application as a quantitative research or monitoring tool within clinically defined populations, particularly in multiple sclerosis, rather than as a standalone screening test for AD, Parkinson disease, or cognitive decline.

### 3.4. Metabolic and Renal Disease

Clinical investigations support the view that retinal signs may indicate the systemic microvascular burden in diabetes. However, just because there are common pathways does not mean the various organ-specific complications are equivalent. Diabetes, hypertension, obesity, and dyslipidemia can disrupt tissue microvascular units before obvious anatomical lesions become apparent; this early dysfunction may remain reversible or progress to anomalies like basement-membrane thickening, capillary rarefaction, and loss of pericytes, with tissue-specific manifestations in the retina, kidney, and peripheral nerves [41].

A meta-analysis of 26 cross-sectional studies estimated the odds of diabetic retinopathy co-occurring with diabetic kidney disease (DKD) and diabetic peripheral neuropathy. The meta-analysis, involving 60,136 participants, found that diabetic retinopathy was significantly associated with diabetic kidney disease (OR, 4.64; 95% CI, 2.47–8.75) and diabetic neuropathy (OR, 2.22; 95% CI, 1.70–2.90) [42]. Thus, diabetic retinopathy may co-occur with other microvascular complications in diabetes. Diabetic retinopathy co-occurred with diabetic kidney disease and diabetic neuropathy. The association with kidney disease strengthened from any diabetic retinopathy (OR, 1.95; 95% CI, 1.62–2.34) to proliferative diabetic retinopathy (OR, 4.44; 95% CI, 2.72–7.24; between-subgroup *p* = 0.002). However, the association of kidney disease with neuropathy was not significant (OR, 1.19; 95% CI, 0.95–1.49). Due to the cross-sectional nature of all included studies, their diagnostic definitions and covariate adjustments varied; heterogeneity was substantial for retinopathy analyses. Thus, these estimates demonstrate co-occurrence, but not directionality or causation. Retinopathy may therefore identify patients requiring evaluation for other microvascular complications, but it cannot be regarded as a surrogate for renal or neurological disease [42].

Evidence targeting the kidneys is wider-ranging but methodologically uneven. Forty-two studies were included following a systematic review strategy: thirty-two were cross-sectional, nine were prospective, and one was retrospective. However, only six studies contributed to four quantitative syntheses. Retinopathy was associated with prevalent chronic kidney disease (CKD) in general populations (OR, 1.31; 95% CI, 1.14–1.50) and with progression to end-stage renal disease (hazard ratio [HR], 2.12; 95% CI, 1.39–3.22), while narrower central retinal arteriolar caliber was associated with prevalent CKD (OR, 1.41; 95% CI, 1.09–1.82). Results collected over time were less consistent. In a cohort of 1256 participants followed for 6 years, narrower arterioles (HR, 1.34; 95% CI, 1.00–1.78), wider venules (HR, 2.35; 95% CI, 1.12–5.94), and retinopathy (HR, 2.54; 95% CI, 1.48–4.36) were associated with incident CKD, while in a cohort of 3302 participants followed for 15 years, retinal vessel caliber was not associated with incident CKD. Retinal vascular signs are, therefore, reflective of contemporaneous renal vascular burden, but the evidence available does not establish a stable prognostic threshold suitable for individual risk stratification [43].

OCT gives more direct structural and longitudinal evidence. In a prospective series that excluded diabetic patients, 112 subjects with CKD had lower macular volume as compared to 86 healthy controls (8.44 ± 0.44 vs. 8.73 ± 0.36 mm^3^; *p* < 0.001) and a thinner subfoveal choroid (274 ± 93 vs. 319 ± 93 μm; *p* < 0.001). Among 50 individuals who underwent kidney biopsy, choroidal thinning at all three measured points remained related to greater interstitial fibrosis and tubular atrophy after adjustment for age, eGFR, glomerulosclerosis, and hypertension, whereas macular volume was not related to the histologic injury. In a longitudinal cohort of 262 patients with CKD, each 10 µm reduction in temporal choroidal thickness (OR, 1.06; 95% CI, 1.01–1.11) and each 1-mm^3^ reduction in macular volume (OR, 2.41; 95% CI, 1.07–5.70) were associated with a ≥10% decline in eGFR at one year. The adjusted associations linked to a ≥20% drop at two years did not reach significance. In a study conducted on 25 renal transplant recipients, choroidal thickness increased by almost 10% over the course of one month, while eGFR increased from 8 ± 3 to 58 ± 21 mL/min/1.73 m^2^ during the first postoperative week. Choroidal thickness remained increased at 12 months. These findings suggest that ocular measurements are dynamic. However, as a result of simultaneous changes in many physiological and treatment determinants post-transplantation, OCT parameters are not yet established as appropriate surrogate endpoints for treatment response [44].

A 2025 meta-analysis of 12 studies including 29,340 participants reinforced the structural signal but also highlighted its limitations: 11 of the 12 studies were cross-sectional. People with CKD had lower average thickness of the peripapillary retinal nerve fiber layer compared with controls, with a standardized mean difference (SMD) of −0.32 (95% CI, −0.44 to −0.20). Similarly, ganglion cell–inner plexiform layer thickness was lower in people with CKD compared with controls, with an SMD of −0.58 (95% CI, −0.78 to −0.38). Finally, macular thickness was reduced in people with CKD, with an SMD of −0.35 (95% CI, −0.58 to −0.12). In contrast, the primary estimate for average choroidal thickness was not statistically significant. Moreover, it was considerably heterogeneous (SMD, −1.84; 95% CI, −4.17 to 0.49; I^2^ = 97.4%); it became significant only after removal of a particular influential study (SMD, −0.66; 95% CI, −0.94 to −0.39), showcasing the instability of this particular measurement across datasets [45].

AI-focused analysis is still early in translation. A fundus-photograph model achieved an area under the receiver operating characteristic curve of 0.911 in internal validation but 0.733 and 0.835 in two external cohorts; conventional risk-factor models performed better in both external datasets (0.829 and 0.887). The image-only model exhibited positive predictive values of merely 14% and 9% and negative predictive values of 97% and 99% in lower-prevalence cohorts. CKD was defined from a single eGFR measurement, albuminuria data were incomplete, and validation was confined to Asian populations. Consequently, retinal AI might support opportunistic first-stage triage followed by conventional renal testing, but its incremental clinical value, effects on patient outcomes, and cost-effectiveness are yet to be established [46].

Another multistage study took this evidence further, targeting DKD instead of broadly targeting CKD. DeepDKD was pretrained on 734,084 fundus photographs, developed and internally validated for DKD detection using 486,312 images from 121,578 people with diabetes, and externally validated on 65,406 people from 10 multiethnic datasets. The image-only system demonstrated an AUC of 0.842 (95% CI, 0.838–0.846) internally; externally, AUCs ranged from 0.791 to 0.826. In a prospective proof-of-concept study involving 325 participants with type 2 diabetes, preserved eGFR and negative urine dipstick protein at baseline, 98 were diagnosed with DKD after elevated albumin-to-creatinine ratios were detected. DeepDKD exhibited a higher sensitivity than a clinical-metadata model (89.8% vs. 66.3%; *p* < 0.0001), but its specificity of 47.6% was not significantly greater than that of the metadata model (38.8%; *p* = 0.072). In order to develop and internally validate the new model for differentiating isolated diabetic nephropathy from non-diabetic kidney disease, 267 biopsy-characterized participants were recruited. The area under the curve (AUC) was 0.906 (95% CI, 0.825–0.966). However, the AUCs fell to between 0.733 and 0.844 in three external datasets comprising 244 participants. In spite of its scale and external validation, this system was trained in a Chinese population, used only gradable photographs, showed heterogeneous performance across cameras and datasets, and underwent only proof-of-concept clinical evaluation. DeepDKD is, therefore, to be understood as an investigational triage tool to prioritize confirmatory laboratory assessment and potentially support nephrological assessment, rather than as a replacement for conventional renal assessment or kidney biopsy [47].

### 3.5. Artificial Intelligence, Screening, and Multimodal Prediction

The translational maturity of retinal AI depends on its intended function. For prognostication, a deep-learning system designed to predict incident mild-or-worse diabetic retinopathy within two years in eyes without baseline retinopathy achieved an area under the receiver operating characteristic curve (AUC) of 0.79 (95% confidence interval [CI], 0.77–0.81) with three-field photography in internal validation and 0.70 (95% CI, 0.67–0.74) with one-field photography in external validation. Combining image-derived predictions with available clinical risk factors increased the AUC from 0.72 to 0.81 internally and from 0.62 to 0.71 externally; however, whether this improvement can safely guide personalized screening intervals has not been tested prospectively [48]. Retrospective diagnostic classification produced higher estimates: a model trained on 128,175 images achieved AUCs of 0.991 and 0.990 in the EyePACS-1 and Messidor-2 datasets, respectively, with sensitivities of 97.5% and 96.1% and specificities of 93.4% and 93.9% at the high-sensitivity operating point [49]. In a larger multiethnic evaluation, the AUC for referable diabetic retinopathy was 0.936, with 90.5% sensitivity and 91.6% specificity in the primary screening dataset; across ten external datasets, AUCs ranged from 0.889 to 0.983. Differences in image quality, cameras, reference standards, and definitions of referability nevertheless preclude direct comparison across datasets [50].

Autonomous implementation was evaluated prospectively in 900 participants attending ten primary-care practices. Of 892 participants completing the protocol, 819 were fully analyzable. The system detected more-than-mild diabetic retinopathy with 87.2% sensitivity (95% CI, 81.8–91.2%) and 90.7% specificity (95% CI, 88.3–92.7%), while producing a disease-level output in 96.1% of participants. Importantly, personnel without previous ocular-imaging experience operated the system after four hours of standardized training, and the workflow incorporated automated image-quality assessment, repeat acquisition, and pharmacological dilation when required [51]. This illustrates why implementation should be evaluated as a complete diagnostic pathway rather than as algorithmic discrimination alone. In a meta-analysis of 34 real-world studies comprising 134,238 participants, pooled sensitivity and specificity among gradable images were 94% (95% CI, 92–96%) and 89% (95% CI, 85–92%), respectively, but substantial heterogeneity remained. Camera type and national income category were associated with diagnostic performance, while patient selection, image inclusion, and the handling of ungradable photographs were frequently insufficiently reported. Performance restricted to gradable images therefore cannot be assumed to represent the effectiveness of an entire screening Program [52].

Systemic image-derived signatures remain less mature. RetiAGE was associated with ten-year morbidity and mortality, but its ability to distinguish individuals aged ≥65 years from those aged <65 years decreased from an AUC of 0.968 internally to 0.756 in the UK cohort, while addition of RetiAGE increased the c-index from 0.706 to 0.720 when added to chronological age and from 0.737 to 0.750 when added to PhenoAGE [53]. Moreover, only good-quality photographs were analyzed, and ethnicity-specific performance could not be assessed. These findings establish an independent prognostic association but do not clarify the biological meaning of the learned signature or its clinical utility [53]. By contrast, the 2026 American Diabetes Association standards support retinal photography with remote interpretation or FDA-approved AI specifically for diabetic retinopathy screening, provided that programs include timely referral, in-person examination for unacceptable images, and comprehensive ophthalmic follow-up when indicated. They do not endorse retinal AI for diagnosing systemic disease [54].

Teleophthalmology may increase the geographical reach of this established screening pathway, but evidence for patient-level benefit remains limited. A review of nine studies involving 322,725 participants found that eight operated in primary care and used trained non-ophthalmic personnel for image acquisition; however, eight were conducted in high-income countries, and the quantitative synthesis estimated diabetic-retinopathy prevalence rather than comparative effects on screening attendance, treatment uptake, visual outcomes, or blindness [55]. Smartphone imaging evidence was also heterogeneous: across nine studies and 1430 participants, pooled sensitivity and specificity for referral-warranted retinopathy were 90.5% (95% CI, 85.5–93.8%) and 88.9% (95% CI, 56.2–98.0%), with substantial heterogeneity. In the two studies using AI to grade smartphone images, sensitivity remained 91.2%, but specificity was only 50.0% [56]. Head-to-head comparisons between commercial algorithms remain scarce, and economic analyses have produced conflicting results across healthcare systems and deployment strategies. Prospective multimodal initiatives such as AI-READI—which plans to combine fundus photography, OCT, OCTA, serological testing, continuous glucose monitoring, electrocardiography, wearable measurements, and social determinants in more than 4000 participants—may improve model development but currently constitute research infrastructure rather than evidence of clinical benefit [57]. The FUTURE-AI consensus translates these limitations into six principles—fairness, universality, traceability, usability, robustness, and explainability—and 30 recommendations covering design, validation, regulation, deployment, and monitoring. Applied to systemic oculomics, this requires prespecifying the target population, clinical action, downstream confirmatory testing, and consequences of false-positive, false-negative, and ungradable results, followed by local validation and continuous post-deployment auditing [58].

### 3.6. Treatment-Response and Precision-Medicine Applications

The most clinically mature precision-medicine application of retinal biomarkers remains the selection and monitoring of ocular treatment, rather than the inference of systemic therapeutic benefit. In diabetic macular edema (DME), OCT is indispensable for follow-up, but persistent DME lacks a standardized definition. A contemporary review proposed assessing response rapidity at week 12 and adequacy at week 24: a <10–25% reduction from baseline central retinal thickness indicates an unsatisfactory anatomical response, no reduction or an increase constitutes treatment failure, and persistence beyond 1 year defines chronic persistent DME. In Protocol T, persistent edema decreased between weeks 12 and 24 from 50.8% to 31.6% with aflibercept, from 72.9% to 65.6% with bevacizumab, and from 53.2% to 41.5% with ranibizumab, demonstrating that an incomplete early response does not necessarily represent a stable treatment-resistant phenotype. Accordingly, the reviewed evidence did not support routine switching during the first year in the absence of treatment failure or early conversion to, or combination with, corticosteroids, except possibly in pseudophakic eyes.

Baseline visual acuity and central retinal thickness remain the principal functional and anatomical indicators, respectively. DRIL and disruption of the ellipsoid zone or external limiting membrane are predominantly associated with poorer visual outcomes, while hyperreflective foci have also been associated with lower subsequent visual acuity, although their relationship with treatment response remains heterogeneous across studies [59,60]. These associations do not necessarily indicate a limited anatomical reduction in macular edema during anti-VEGF therapy. Accordingly, these OCT features are better regarded as candidate prognostic biomarkers of visual outcome than as validated predictive biomarkers for treatment selection, and they do not establish that an alternative treatment would provide greater benefit.

Microglial regulation provides a mechanistic basis for more targeted strategies, but the supporting evidence remains predominantly preclinical. In streptozotocin-induced diabetic rats, reduced retinal blood flow after 4 weeks of diabetes coincided with selective narrowing of superficial-plexus capillaries, increased microglial–capillary contact, loss of vascular responses to hyperoxia and fractalkine, and increased microglial angiotensinogen expression. These findings implicate fractalkine–CX3CR1 and renin–angiotensin signaling in early microglia-dependent vasoconstriction. Nevertheless, retinal vasoregulation has principally been demonstrated in explant models, and current label-free human adaptive-optics imaging cannot reliably identify intraretinal microglia. Microglial activity therefore remains a plausible therapeutic target rather than a clinically validated response biomarker [61].

EUROCONDOR illustrates the evidentiary threshold required for biomarker-guided treatment. In this randomized, placebo-controlled phase II–III trial, 449 adults with type 2 diabetes and no or mild retinopathy received brimonidine 0.2% (*n* = 152), somatostatin 0.1% (*n* = 145), or placebo (*n* = 152) twice daily for 96 weeks. Neither active treatment improved the whole-cohort primary outcome of multifocal electroretinographic implicit time. Among the 156 participants (34.7%) with neurodysfunction at baseline, a prespecified per-protocol analysis showed worsening with placebo (*p* < 0.001) but stability with both active treatments; however, the corresponding intention-to-treat analysis did not reach statistical significance (*p* = 0.06). Neither drug altered microvascular progression or OCT-derived retinal thickness, and attrition reached 24%. Baseline neurodysfunction can therefore be considered a candidate biomarker for trial enrichment and further evaluation as a predictive biomarker, but not an established determinant of treatment benefit or a validated surrogate endpoint for microvascular—still less systemic—disease modification [62]. The principal retinal biomarker applications and their current translational status are summarized in Table 1.

## 4. Discussion

The principal finding of this review is that the retina contains measurable information about systemic vascular, neural, and metabolic injury, but that this information does not constitute a single, universally applicable systemic biomarker. Translational maturity is specific to the modality, target disease, intended population, and clinical function. Autonomous diabetic-retinopathy screening [51,54] and OCT-guided management of retinal disease [59] have entered defined clinical pathways. Retinal vascular signs have longitudinal epidemiological validity for cardiovascular and cerebrovascular outcomes, although associations vary by vascular parameter, outcome, sex, and analytical model [23,24]. In multiple sclerosis, OCT now has an adjunctive diagnostic role in addition to its established value for neuroaxonal assessment: under the 2024 McDonald revisions, OCT evidence of optic nerve injury can contribute to dissemination in space within an appropriate neurological assessment, although OCT does not independently establish the diagnosis of multiple sclerosis [37,38]. Conversely, standalone retinal diagnosis of Alzheimer or Parkinson disease [31,32,36], individual OCTA thresholds for systemic disease [13,16,33,40], and retinal AI prediction of cardiovascular or renal outcomes remain investigational [28,46,47]. The retina should therefore be considered an accessible measurement site whose relevance must be established separately for each proposed use, rather than an organ-independent surrogate for systemic disease.

To preserve these distinctions, we propose a five-domain translational framework. The descriptors used throughout this review, including ‘established,’ ‘more clinically mature,’ and ‘investigational,’ represent a qualitative synthesis by the authors informed by these five domains and the characteristics of the available evidence; they do not constitute a formally validated grading or evidence-ranking system. Analytical validity concerns whether the retinal feature can be measured reproducibly under specified acquisition, device, segmentation, and quality-control conditions. Clinical validity requires an association with the intended condition or outcome in the target population, using an appropriate reference standard. Incremental value asks whether the retinal marker improves prediction or classification beyond information already available in routine care. Clinical utility requires evidence that acting on the result changes management or improves patient-relevant outcomes. Implementation additionally depends on feasibility, cost, acceptability, equity, interpretability, data governance, and the capacity to complete downstream assessment and treatment. These domains should not be assigned globally to an imaging modality. The same fundus photograph can support regulated diabetic-retinopathy screening [51,54] while providing only retrospectively validated evidence for systemic risk prediction [28,46]; similarly, OCT can guide treatment of diabetic macular edema [59] without being sufficiently accurate for Alzheimer-disease diagnosis [31,32]. Most systemic oculomics studies reviewed here address clinical association or discrimination, whereas incremental value, clinical utility, and implementation have rarely been demonstrated [3,4,7]. The evidentiary requirements and principal unresolved gaps within each translational domain are summarized in Table 2.

Analytical validity is the first major constraint because a retinal biomarker is defined not only by the biological feature but also by the process used to generate it. OCTA metrics represent detected motion contrast rather than direct volumetric blood flow and vary with scan dimensions, segmentation boundaries, thresholding, projection correction, signal strength, and the lower limit of detectable flow. Differences between devices and analytical pipelines can therefore produce numerically different values for nominally similar measures [12,13]. Structural OCT is more standardized, but reporting still requires specification of the device, acquisition protocol, measurement area, segmentation procedure, and manual correction, as emphasized by APOSTEL 2.0 [14]. Fundus-derived vessel caliber is similarly dependent on image field, vessel-selection rules, magnification correction, and software implementation. Excluding ungradable images does not merely improve a dataset: it changes the population in which performance is estimated and can overstate pathway-level effectiveness when image failure is not reported [52]. Until acquisition and analysis are sufficiently harmonized, universal thresholds and direct comparison of results across studies are not defensible.

Clinical interpretation is further limited by the fact that retinal abnormalities may have several non-equivalent meanings. They may reflect shared exposure to aging, hypertension, hyperglycemia, smoking, or renal dysfunction; accumulated end-organ injury; local ocular pathology; a mediator of systemic disease; or a correlate without a causal role. Longitudinal cardiovascular studies establish that some retinal changes precede events, but temporality alone does not show that modifying the retinal feature would alter cardiovascular risk [18,19,20,21,22,23,24]. The weaker and less consistent findings obtained when Alzheimer disease is defined by cerebral amyloid status rather than by clinical diagnosis demonstrate how strongly apparent retinal validity depends on the reference standard and case definition [31,32]. Conversely, the larger and more consistent OCT differences in multiple sclerosis remain dependent on optic-neuritis history and do not make retinal thinning disease-specific [37]. In CKD, the association of retinal and choroidal measurements with subsequent eGFR decline supports temporal relevance, whereas choroidal thickening after kidney transplantation also shows that ocular measurements respond to rapidly changing hemodynamic, fluid, and therapeutic conditions [44]. Physiological responsiveness should therefore not be equated with specificity for renal recovery or validation as a treatment surrogate.

Incremental value represents the point at which many otherwise promising biomarkers become less persuasive. Retinal models can identify cardiovascular or renal signals, but comparisons with established clinical predictors have shown improvements that are modest, absent, or dependent on the evaluated population and fitted baseline model [25,27,28,46,53]. Reclassification is clinically meaningful only when it changes an appropriate decision and produces more benefit than harm. None of the systemic applications included in this review demonstrated through a prospective impact study that retinal-guided cardiovascular, neurological, or renal management improved clinical outcomes. The prospective autonomous diabetic retinopathy trial is an important exception because it evaluated a specified target condition, operating threshold, acquisition workflow, image-quality procedure, and referral pathway [51]. Its success validates that particular ocular use case; it cannot be extrapolated to systemic predictions generated from the same type of image. Likewise, OCT outcomes in multiple sclerosis and diabetic macular edema are supported within their respective disease and treatment contexts but should not be interpreted as validated population-screening or systemic-surrogate measures [37,59].

AI increases the amount of information recoverable from an image but does not remove these evidentiary requirements. The reduction in performance observed between internal and external datasets for diabetic-retinopathy prediction, CKD detection, and retinal biological age demonstrates sensitivity to population and dataset differences [46,48,53]. Real-world diabetic-retinopathy studies further show that camera type, selection procedures, image gradability, and healthcare setting affect diagnostic performance [52]. Evaluation should therefore reproduce the intended disease prevalence, report calibration and prespecified subgroup performance, retain ungradable examinations in pathway-level analyses, and define how missing or poor-quality data are managed. The relevant intervention is the complete AI-assisted pathway: acquisition, quality assessment, model output, result communication, confirmatory testing, referral, and follow-up. The FUTURE-AI principles of fairness, universality, traceability, usability, robustness, and explainability provide a coherent structure for designing and monitoring that pathway [58]. Explainability may facilitate auditing and reveal implausible dependencies, but it cannot compensate for inadequate external validation, poor calibration, or absence of clinical benefit.

The most defensible near-term model for systemic oculomics is therefore adjunctive and hypothesis-driven. Images acquired for indicated ophthalmic care could be used to identify individuals for conventional systemic assessment, but the target population, confirmatory test, action threshold, and downstream responsibility must be defined prospectively. Such a pathway would test whether retinal information improves existing assessment rather than treating an image-derived score as an independent diagnosis. This distinction is especially important in neurological disease, where retinal thinning and microvascular loss occur across multiple disorders and should be evaluated as components of multimodal models incorporating appropriate clinical, cognitive, laboratory, genetic, or neuroimaging information [31,32,33,34,35,36,37,38,39,40]. Any opportunistic program must additionally address consent for secondary analysis, communication of uncertain or incidental risk, data protection, and responsibility for ensuring that an abnormal result receives appropriate confirmation [3,4,58].

Repeated imaging may ultimately be more informative than a single measurement, but longitudinal change requires equally careful interpretation. Within-person trajectories may reduce some between-person variability and can test whether retinal features respond to changing systemic physiology, as suggested by the CKD and transplantation data [44]. Nevertheless, the treatment literature shows why responsiveness is not equivalent to surrogacy. In persistent diabetic macular edema, anatomical response depends on assessment time and treatment exposure, and proposed OCT predictors do not yet constitute universally validated rules for switching treatment [59]. In EUROCONDOR, baseline neurodysfunction identified a potentially responsive subgroup in the per-protocol analysis, but the whole-cohort endpoint was negative, the corresponding intention-to-treat subgroup result was not statistically significant, and no effect on microvascular progression was demonstrated [62]. Future studies must consequently distinguish prognostic biomarkers, which identify the likelihood of future outcomes or disease progression; predictive biomarkers, which identify differential treatment effects; pharmacodynamic biomarkers, which indicate a biological response following an intervention; and surrogate endpoints, which would need to reliably predict a clinically meaningful treatment effect. The next research phase should be organized around prespecified clinical uses rather than further undirected associations. Multicentre studies should include ethnically and clinically diverse populations, multiple imaging devices, common ocular comorbidities, and participants with ungradable or imperfect images. Acquisition and segmentation protocols, reference standards, eligibility criteria, endpoints, thresholds, and analysis plans should be defined before model evaluation. External validation should be geographically and temporally independent and should assess calibration, discrimination, subgroup performance, and failure rates against the same conventional information available in the intended pathway. Prospective comparative studies should then determine whether adding the retinal biomarker changes appropriate referrals, diagnostic yield, treatment initiation, patient-important outcomes, workload, equity, and cost. Transparent version control and post-deployment monitoring are necessary because changes in cameras, software, segmentation, or clinical populations can alter performance after implementation [3,13,14,52,57,58].

There are limitations to this review. The narrative design allowed integration of heterogeneous cardiovascular, neurological, metabolic, renal, imaging, and AI evidence but did not allow for exhaustive retrieval, duplicate study selection, formal study-level risk-of-bias assessment, certainty grading, or a new pooled estimate. The search was restricted to PubMed and English-language peer-reviewed literature; consequently, relevant studies indexed exclusively in other bibliographic databases may have been missed. Interpretive selection was also necessary because landmark studies and high-level evidence were prioritized. Substantial changes in imaging software, the AI models used, and their regulatory status may impact the future applicability of individual findings. Thus, the current synthesis should be regarded as a critical framework for assessing retinal biomarkers and shaping translational studies, rather than a guideline permitting systemic diagnosis or treatment decisions based on retinal images.

## 5. Conclusions

Retinal imaging combines non-invasive accessibility, direct visualization of the microvasculature, quantitative assessment of neural tissue, and compatibility with scalable digital analysis. Current evidence supports established ophthalmic applications and demonstrates that retinal phenotypes are associated with cardiovascular, cerebrovascular, neurological, metabolic, and renal diseases and outcomes. However, the strength and translational maturity of these associations vary substantially across biomarkers and intended uses. The principal priority is therefore not the identification of further associations alone but analytical standardization, independent validation in the intended clinical setting, demonstration of incremental value over established predictors, and prospective evaluation of whether retinal-biomarker–informed decisions improve patient-relevant outcomes. Diabetes provides the most developed translational model because diabetic-retinopathy screening and OCT-guided ocular treatment monitoring are established clinical applications, whereas retinal AI for systemic-disease prediction remains investigational. Even in diabetes, however, a biomarker validated for ocular detection, prognosis, or treatment response cannot be assumed to diagnose, monitor, or serve as a surrogate for systemic disease. If prospectively validated, the most plausible near-term role of systemic retinal biomarkers is therefore adjunctive, within prespecified pathways that incorporate conventional assessment and confirmatory testing. Such pathways must also be evaluated for calibration across populations, fairness, downstream workload and harms, follow-up capacity, and cost-effectiveness. Until clinical utility is demonstrated, most systemic applications of retinal imaging should remain investigational rather than being used as standalone diagnostic or treatment-guiding tests.

## Figures and Tables

**Table 1:** Current translational status of the principal retinal biomarker applications reviewed.

Clinical Application	Principal Retinal Input	Level of Evidence Supported	Current Translational Interpretation
Diabetic-retinopathy screening	Color fundus photography; autonomous AI	Individual-level prediction and clinical decision-making supported by prospective and real-world diagnostic evidence	Established ocular screening pathway; requires appropriate management of ungradable images, referral, and follow-up [51,52,54]
Diabetic retinal treatment response	Structural OCT; mfERG	Individual-level treatment monitoring; candidate predictive evidence remains exploratory	OCT supports ocular management; predictive-biomarker and surrogate-endpoint roles remain unproven [59,62]
Cardiovascular and cerebrovascular disease	Retinal vascular signs and caliber; image-derived AI scores	Population-level association; individual-level prediction shows modest incremental discrimination in selected models	Population-level validity; clinical benefit from retinal-guided care has not been demonstrated [23,24,28]
Multiple sclerosis	OCT-derived pRNFL and GCIPL thickness	Group-level and longitudinal monitoring evidence; defined OCT inter-eye differences can support evidence of optic nerve involvement within the 2024 McDonald diagnostic framework	Clinical adjunct within established neurological assessment and monitoring; not a standalone screening or diagnostic test [37,38]
Alzheimer disease, Parkinson disease, and cognitive decline	OCT, OCTA, and fundus microvascular measures	Population-level/group-level association; individual-level diagnostic prediction remains insufficiently validated	Investigational; requires biomarker-defined and prospective validation [31,32,33,36,40]
Chronic and diabetic kidney disease	Retinal vascular signs, retinal and choroidal OCT, and fundus-based AI	Predominantly population-level association; individual-level prediction remains investigational	Investigational adjunctive triage; confirmatory laboratory and nephrological assessment remain necessary [44,45,46,47]

AI, artificial intelligence; GCIPL, ganglion cell–inner plexiform layer; mfERG, multifocal electroretinography; OCT, optical coherence tomography; OCTA, optical coherence tomography angiography; pRNFL, peripapillary retinal nerve fiber layer. Translational status refers to the specific biomarker–use-case combination and represents a qualitative synthesis based on the five translational domains described in the Discussion; it is not a formally validated grading system and should not be extrapolated to other diseases or clinical functions.

**Table 2:** Five-domain framework for the clinical translation of retinal biomarkers.

Translational Domain	Core Question	Evidence Required	Predominant Gap in the Reviewed Literature
Analytical validity	Can the feature be measured reproducibly?	Standardized acquisition, repeatability, segmentation, quality control, and explicit handling of ungradable images	Device- and pipeline-dependent measurements, particularly for OCTA [12,13,14,52]
Clinical validity	Is the marker related to the intended disease or outcome in the target population?	Appropriate reference standard and independent, preferably longitudinal, validation	Cross-sectional designs, heterogeneous case definitions, confounding, and limited individual-level accuracy [31,32,43,45]
Incremental value	Does the marker improve existing clinical assessment?	Direct comparison with a prespecified conventional model, including calibration and risk classification	Improvement is frequently modest, population-dependent, or not evaluated [25,28,46,53]
Clinical utility	Does acting on the result improve decisions or outcomes?	Prospective comparative studies assessing management, patient outcomes, and potential harms	Prospective pathway evidence is largely confined to diabetic retinopathy screening [51,54]
Implementation	Can the pathway operate safely, equitably, and sustainably?	Multisite and multidevice evaluation, defined referral and follow-up, fairness, cost assessment, governance, and post-deployment monitoring	Limited transportability, inconsistent handling of image failure, and uncertain downstream capacity [52,57,58]

## Data Availability

No new data were created or analyzed in this study. Data sharing is not applicable to this article.

## References

[1] NCD Risk Factor Collaboration (NCD-RisC) (2024). Worldwide trends in diabetes prevalence and treatment from 1990 to 2022: A pooled analysis of 1108 population-representative studies with 141 million participants. Lancet.

[2] Teo Z.L., Tham Y.C., Yu M., Chee M.L., Rim T.H., Cheung N., Bikbov M.M., Wang Y.X., Tang Y., Lu Y. (2021). Global Prevalence of Diabetic Retinopathy and Projection of Burden through 2045: Systematic Review and Meta-analysis. Ophthalmology.

[3] Chew E.Y., Burns S.A., Abraham A.G., Bakhoum M.F., Beckman J.A., Chui T.Y.P., Finger R.P., Frangi A.F., Gottesman R.F., Grant M.B. (2025). Standardization and clinical applications of retinal imaging biomarkers for cardiovascular disease: A Roadmap from an NHLBI workshop. Nat. Rev. Cardiol..

[4] Zhu Z., Wang Y., Qi Z., Hu W., Zhang X., Wagner S.K., Wang Y., Ran A.R., Ong J., Waisberg E. (2025). Oculomics: Current concepts and evidence. Prog. Retin. Eye Res..

[5] Wagner S.K., Fu D.J., Faes L., Liu X., Huemer J., Khalid H., Ferraz D., Korot E., Kelly C., Balaskas K. (2020). Insights into Systemic Disease through Retinal Imaging-Based Oculomics. Transl. Vis. Sci. Technol..

[6] London A., Benhar I., Schwartz M. (2013). The retina as a window to the brain-from eye research to CNS disorders. Nat. Rev. Neurol..

[7] Wang J., Wang Y.X., Zeng D., Zhu Z., Li D., Liu Y., Sheng B., Grzybowski A., Wong T.Y. (2025). Artificial intelligence-enhanced retinal imaging as a biomarker for systemic diseases. Theranostics.

[8] Baethge C., Goldbeck-Wood S., Mertens S. (2019). SANRA-a scale for the quality assessment of narrative review articles. Res. Integr. Peer Rev..

[9] Wong T.Y., Cheung C.M., Larsen M., Sharma S., Simó R. (2016). Diabetic retinopathy. Nat. Rev. Dis. Primers.

[10] Antonetti D.A., Silva P.S., Stitt A.W. (2021). Current understanding of the molecular and cellular pathology of diabetic retinopathy. Nat. Rev. Endocrinol..

[11] Stitt A.W., Curtis T.M., Chen M., Medina R.J., McKay G.J., Jenkins A., Gardiner T.A., Lyons T.J., Hammes H.P., Simó R. (2016). The progress in understanding and treatment of diabetic retinopathy. Prog. Retin. Eye Res..

[12] Waheed N.K., Rosen R.B., Jia Y., Munk M.R., Huang D., Fawzi A., Chong V., Nguyen Q.D., Sepah Y., Pearce E. (2023). Optical coherence tomography angiography in diabetic retinopathy. Prog. Retin. Eye Res..

[13] Courtie E., Kirkpatrick J.R.M., Taylor M., Faes L., Liu X., Logan A., Veenith T., Denniston A.K., Blanch R.J. (2024). Optical coherence tomography angiography analysis methods: A systematic review and meta-analysis. Sci. Rep..

[14] Aytulun A., Cruz-Herranz A., Aktas O., Balcer L.J., Balk L., Barboni P., Blanco A.A., Calabresi P.A., Costello F., Sanchez-Dalmau B. (2021). APOSTEL 2.0 Recommendations for Reporting Quantitative Optical Coherence Tomography Studies. Neurology.

[15] Tang Z., Chan M.Y., Leung W.Y., Wong H.Y., Ng C.M., Chan V.T.T., Wong R., Lok J., Szeto S., Chan J.C.K. (2021). Assessment of retinal neurodegeneration with spectral-domain optical coherence tomography: A systematic review and meta-analysis. Eye.

[16] Zhang B., Chou Y., Zhao X., Yang J., Chen Y. (2021). Early Detection of Microvascular Impairments with Optical Coherence Tomography Angiography in Diabetic Patients Without Clinical Retinopathy: A Meta-analysis. Am. J. Ophthalmol..

[17] Parravano M., Cennamo G., Di Antonio L., Grassi M.O., Lupidi M., Rispoli M., Savastano M.C., Veritti D., Vujosevic S. (2024). Multimodal imaging in diabetic retinopathy and macular edema: An update about biomarkers. Surv. Ophthalmol..

[18] Wong T.Y., Klein R., Couper D.J., Cooper L.S., Shahar E., Hubbard L.D., Wofford M.R., Sharrett A.R. (2001). Retinal microvascular abnormalities and incident stroke: The Atherosclerosis Risk in Communities Study. Lancet.

[19] Wong T.Y., Klein R., Sharrett A.R., Duncan B.B., Couper D.J., Tielsch J.M., Klein B.E., Hubbard L.D. (2002). Retinal arteriolar narrowing and risk of coronary heart disease in men and women. The Atherosclerosis Risk in Communities Study. JAMA.

[20] McGeechan K., Liew G., Macaskill P., Irwig L., Klein R., Klein B.E., Wang J.J., Mitchell P., Vingerling J.R., Dejong P.T. (2009). Meta-analysis: Retinal vessel caliber and risk for coronary heart disease. Ann. Intern. Med..

[21] McGeechan K., Liew G., Macaskill P., Irwig L., Klein R., Klein B.E., Wang J.J., Mitchell P., Vingerling J.R., de Jong P.T. (2009). Prediction of incident stroke events based on retinal vessel caliber: A systematic review and individual-participant meta-analysis. Am. J. Epidemiol..

[22] Ding J., Wai K.L., McGeechan K., Ikram M.K., Kawasaki R., Xie J., Klein R., Klein B.B., Cotch M.F., Wang J.J. (2014). Meta-Eye Study Group. Retinal vascular caliber and the development of hypertension: A meta-analysis of individual participant data. J. Hypertens..

[23] Seidelmann S.B., Claggett B., Bravo P.E., Gupta A., Farhad H., Klein B.E., Klein R., Di Carli M., Solomon S.D. (2016). Retinal Vessel Calibers in Predicting Long-Term Cardiovascular Outcomes: The Atherosclerosis Risk in Communities Study. Circulation.

[24] Guo S., Yin S., Tse G., Li G., Su L., Liu T. (2020). Association Between Caliber of Retinal Vessels and Cardiovascular Disease: A Systematic Review and Meta-Analysis. Curr. Atheroscler. Rep..

[25] Poplin R., Varadarajan A.V., Blumer K., Liu Y., McConnell M.V., Corrado G.S., Peng L., Webster D.R. (2018). Prediction of cardiovascular risk factors from retinal fundus photographs via deep learning. Nat. Biomed. Eng..

[26] Cheung C.Y., Xu D., Cheng C.Y., Sabanayagam C., Tham Y.C., Yu M., Rim T.H., Chai C.Y., Gopinath B., Mitchell P. (2021). A deep-learning system for the assessment of cardiovascular disease risk via the measurement of retinal-vessel calibre. Nat. Biomed. Eng..

[27] Rim T.H., Lee C.J., Tham Y.C., Cheung N., Yu M., Lee G., Kim Y., Ting D.S.W., Chong C.C.Y., Choi Y.S. (2021). Deep-learning-based cardiovascular risk stratification using coronary artery calcium scores predicted from retinal photographs. Lancet Digit. Health.

[28] Tseng R.M.W.W., Rim T.H., Shantsila E., Yi J.K., Park S., Kim S.S., Lee C.J., Thakur S., Nusinovici S., Peng Q. (2023). Validation of a deep-learning-based retinal biomarker (Reti-CVD) in the prediction of cardiovascular disease: Data from UK Biobank. BMC Med..

[29] Heringa S.M., Bouvy W.H., van den Berg E., Moll A.C., Kappelle L.J., Biessels G.J. (2013). Associations between retinal microvascular changes and dementia, cognitive functioning, and brain imaging abnormalities: A systematic review. J. Cereb. Blood Flow Metab..

[30] Cheung C.Y., Ikram M.K., Chen C., Wong T.Y. (2017). Imaging retina to study dementia and stroke. Prog. Retin. Eye Res..

[31] Costanzo E., Lengyel I., Parravano M., Biagini I., Veldsman M., Badhwar A., Betts M., Cherubini A., Llewellyn D.J., Lourida I. (2023). Ocular Biomarkers for Alzheimer Disease Dementia: An Umbrella Review of Systematic Reviews and Meta-analyses. JAMA Ophthalmol..

[32] Ashraf G., McGuinness M., Khan M.A., Obtinalla C., Hadoux X., van Wijngaarden P. (2023). Retinal imaging biomarkers of Alzheimer’s disease: A systematic review and meta-analysis of studies using brain amyloid beta status for case definition. Alzheimers Dement..

[33] Yeh T.C., Kuo C.T., Chou Y.B. (2022). Retinal Microvascular Changes in Mild Cognitive Impairment and Alzheimer’s Disease: A Systematic Review, Meta-Analysis, and Meta-Regression. Front. Aging Neurosci..

[34] Leal-Bernal C., Noriega-Ramírez S., Álvarez-Martínez J.V., Cifuentes-González C., Rojas-Carabali W., Manrique-Samer A., Flórez-Esparza V., Monsalve-García I., Amézquita-Villanueva J.S., Mejía-Salgado G. (2025). Retinal and Vascular Findings in Optical Coherence Tomography in Healthy Cognitive Patients with Alzheimer Disease Biomarkers: A Systematic Review and Meta-Analysis. Alzheimer Dis. Assoc. Disord..

[35] Hao J., Kwapong W.R., Shen T., Fu H., Xu Y., Lu Q., Liu S., Zhang J., Liu Y., Zhao Y. (2024). Early detection of dementia through retinal imaging and trustworthy AI. NPJ Digit. Med..

[36] Zhou W.C., Tao J.X., Li J. (2021). Optical coherence tomography measurements as potential imaging biomarkers for Parkinson’s disease: A systematic review and meta-analysis. Eur. J. Neurol..

[37] Petzold A., Balcer L.J., Calabresi P.A., Costello F., Frohman T.C., Frohman E.M., Martinez-Lapiscina E.H., Green A.J., Kardon R., Outteryck O. (2017). Retinal layer segmentation in multiple sclerosis: A systematic review and meta-analysis. Lancet Neurol..

[38] Saidha S., Green A.J., Leocani L., Vidal-Jordana A., Kenney R.C., Bsteh G., Outteryck O., Thompson A., Montalban X., Coetzee T. (2025). The use of optical coherence tomography and visual evoked potentials in the 2024 McDonald diagnostic criteria for multiple sclerosis. Lancet Neurol..

[39] Ko F., Muthy Z.A., Gallacher J., Sudlow C., Rees G., Yang Q., Keane P.A., Petzold A., Khaw P.T., Reisman C. (2018). Association of Retinal Nerve Fiber Layer Thinning with Current and Future Cognitive Decline: A Study Using Optical Coherence Tomography. JAMA Neurol..

[40] Alruwais N., Althobaiti G.A., Alaql Y.B., Almutairi S.A.H., Alshareef W.M., Mahrous F.A., Aloqaybi S.A.S., Lasloom R.A.A., Aljasser A.A. (2026). Retinal Microvascular Biomarkers Measured by Optical Coherence Tomography Angiography in Neurodegenerative and Neurovascular Disorders: A Systematic Review and Meta-Analysis. Clin. Ophthalmol..

[41] Horton W.B., Barrett E.J. (2021). Microvascular Dysfunction in Diabetes Mellitus and Cardiometabolic Disease. Endocr. Rev..

[42] Li J., Cao Y., Liu W., Wang Q., Qian Y., Lu P. (2019). Correlations among Diabetic Microvascular Complications: A Systematic Review and Meta-analysis. Sci. Rep..

[43] Aronov M., Allon R., Stave D., Belkin M., Margalit E., Fabian I.D., Rosenzweig B. (2021). Retinal Vascular Signs as Screening and Prognostic Factors for Chronic Kidney Disease: A Systematic Review and Meta-Analysis of Current Evidence. J. Pers. Med..

[44] Farrah T.E., Pugh D., Chapman F.A., Godden E., Balmforth C., Oniscu G.C., Webb D.J., Dhillon B., Dear J.W., Bailey M.A. (2023). Choroidal and retinal thinning in chronic kidney disease independently associate with eGFR decline and are modifiable with treatment. Nat. Commun..

[45] Bineshfar N., Changizi F., Farjam M., Sharafi F., Williams B.K. (2025). Systematic review and meta-analysis of optical coherence tomography measurements in patients with chronic kidney disease. Retina.

[46] Sabanayagam C., Xu D., Ting D.S.W., Nusinovici S., Banu R., Hamzah H., Lim C., Tham Y.C., Cheung C.Y., Tai E.S. (2020). A deep learning algorithm to detect chronic kidney disease from retinal photographs in community-based populations. Lancet Digit. Health.

[47] Meng Z., Guan Z., Yu S., Wu Y., Zhao Y., Shen J., Lim C.C., Chen T., Yang D., Ran A.R. (2025). Non-invasive biopsy diagnosis of diabetic kidney disease via deep learning applied to retinal images: A population-based study. Lancet Digit. Health.

[48] Bora A., Balasubramanian S., Babenko B., Virmani S., Venugopalan S., Mitani A., de Oliveira Marinho G., Cuadros J., Ruamviboonsuk P., Corrado G.S. (2021). Predicting the risk of developing diabetic retinopathy using deep learning. Lancet Digit. Health.

[49] Gulshan V., Peng L., Coram M., Stumpe M.C., Wu D., Narayanaswamy A., Venugopalan S., Widner K., Madams T., Cuadros J. (2016). Development and Validation of a Deep Learning Algorithm for Detection of Diabetic Retinopathy in Retinal Fundus Photographs. JAMA.

[50] Ting D.S.W., Cheung C.Y., Lim G., Tan G.S.W., Quang N.D., Gan A., Hamzah H., Garcia-Franco R., San Yeo I.Y., Lee S.Y. (2017). Development and Validation of a Deep Learning System for Diabetic Retinopathy and Related Eye Diseases Using Retinal Images from Multiethnic Populations with Diabetes. JAMA.

[51] Abràmoff M.D., Lavin P.T., Birch M., Shah N., Folk J.C. (2018). Pivotal trial of an autonomous AI-based diagnostic system for detection of diabetic retinopathy in primary care offices. NPJ Digit. Med..

[52] Joseph S., Selvaraj J., Mani I., Kumaragurupari T., Shang X., Mudgil P., Ravilla T., He M. (2024). Diagnostic Accuracy of Artificial Intelligence-Based Automated Diabetic Retinopathy Screening in Real-World Settings: A Systematic Review and Meta-Analysis. Am. J. Ophthalmol..

[53] Nusinovici S., Rim T.H., Yu M., Lee G., Tham Y.C., Cheung N., Chong C.C.Y., Da Soh Z., Thakur S., Lee C.J. (2022). Retinal photograph-based deep learning predicts biological age, and stratifies morbidity and mortality risk. Age Ageing.

[54] American Diabetes Association Professional Practice Committee for Diabetes (2026). 12. Retinopathy, Neuropathy, and Foot Care: Standards of Care in Diabetes-2026. Diabetes Care.

[55] Zikhali T., Kalinda C., Xulu-Kasaba Z.N. (2022). Screening of Diabetic Retinopathy Using Teleophthalmology to Complement Human Resources for Eye Health: A Systematic Review and Meta-Analysis. Clin. Pract..

[56] Tan C.H., Kyaw B.M., Smith H., Tan C.S., Tudor Car L. (2020). Use of Smartphones to Detect Diabetic Retinopathy: Scoping Review and Meta-Analysis of Diagnostic Test Accuracy Studies. J. Med. Internet Res..

[57] Rajesh A.E., Davidson O.Q., Lee C.S., Lee A.Y. (2023). Artificial Intelligence and Diabetic Retinopathy: AI Framework, Prospective Studies, Head-to-head Validation, and Cost-effectiveness. Diabetes Care.

[58] Lekadir K., Frangi A.F., Porras A.R., Glocker B., Cintas C., Langlotz C.P., Weicken E., Asselbergs F.W., Prior F., Collins G.S. (2025). FUTURE-AI: International consensus guideline for trustworthy and deployable artificial intelligence in healthcare. BMJ.

[59] Sorour O.A., Levine E.S., Baumal C.R., Elnahry A.G., Braun P., Girgis J., Waheed N.K. (2023). Persistent diabetic macular edema: Definition, incidence, biomarkers, and treatment methods. Surv. Ophthalmol..

[60] Nanji K., Grad J., Hatamnejad A., El-Sayes A., Mihalache A., Gemae M., Huang R., Phillips M., Kaiser P.K., Munk M.R. (2026). Baseline OCT Biomarkers Associated with Visual Acuity in Diabetic Macular Edema: A Systematic Review and Meta-analysis. Ophthalmology.

[61] Jobling A.I., Greferath U., Dixon M.A., Quiriconi P., Eyar B., van Koeverden A.K., Mills S.A., Vessey K.A., Bui B.V., Fletcher E.L. (2025). Microglial regulation of the retinal vasculature in health and during the pathology associated with diabetes. Prog. Retin. Eye Res..

[62] Simó R., Hernández C., Porta M., Bandello F., Grauslund J., Harding S.P., Aldington S.J., Egan C., Frydkjaer-Olsen U., García-Arumí J. (2019). Effects of Topically Administered Neuroprotective Drugs in Early Stages of Diabetic Retinopathy: Results of the EUROCONDOR Clinical Trial. Diabetes.

